# The Iodine Method of Skin Sterilisation

**Published:** 1914-11-21

**Authors:** 


### THE IODINE METHOD OF SKIN STERILISATION.

### Its Value against Septic Infection.

Everyone whose surgical experience runs back as far as five or six years will acknowledge that the methods then usual for securing the (comparative) sterilisation of the skin at the site of an intended operation were complicated and productive of very considerable discomfort to the patient. As a general rule the preparation began twelve, or even twenty-four, hours before the time fixed for the visit to the theatre. It began with shaving, when necessary, followed by hard scrubbing with nail-brush and soap for some minutes. The reddened irritated surface thus produced was then freely swabbed with ether or turpentine: the former produced a sensation of icy cold, the latter tended to aggravate irritation, and either was distinctly uncomfortable. Then came a free use of some antiseptic, usually biniodide of mercury or carbolic acid lotion; after which the process was completed by a fomentation wrung out of the same fluid. This fomentation remained on all through the night previous to the operation, and its sodden weight was not exactly calculated to soothe the natural sleeplessness of a sensitive individual.

Nowadays the iodine method is in favour. It is vastly simpler, entails no discomfort, and is said by those who introduced it to be safer than the more barbarous old-fashioned methods. It was originally propounded by Grossich. Its widespread popularity may be interpreted as convincing evidence of its efficiency, since failure to produce a reasonable degree of asepsis by its use would soon be apparent to surgeons employing it. Such a deduction is not really safe, for the obvious reason that any surgeon faced by a case of operative sepsis arising in a clean case has to consider so many possible portals of entry of the septic micro-organisms. Nor is it true that surgeons are entirely unanimous in their agreement as to the virtues of the iodine method. In this country there are several doubters, though their voices have not hitherto been much heard. In this connection it is significant that, at the recent Congress of Surgeons, Mr. Tubby warned his hearers against being satisfied with the iodine method when contemplating the opening of the knee-joint for the removal of a loose cartilage within it. Suppuration is so disastrous in knee-joint operations, and the joint seems to be so susceptible to septic infection, that this indictment of the iodine method seems to suggest that it does not give the same degree of security obtainable by the older methods.

In a recent paper* the matter has been considered very thoroughly from the experimental standpoint by Dr. J. W. Bovee, of Washington. This observer has long contended that the 2-per-cent. iodine solution which satisfies most British surgeons is not strong enough, and that a 3½-per-cent. solution in 95-per-cent. alcohol is required to ensure complete sterilisation of the skin. He quotes a statement by another American surgeon (Robb) to the effect that bacteriological tests show that the iodine method of skin preparation "is not to be relied on, and should only be used when more elaborate forms of sterilisation are contra-indicated." But he acknowledges that the Mayo Clinic and many other first-rate clinics in America are using the method with success, and he is now investigating the apparent conflict between bacteriological evidence and practical surgical experience.

The skin of ten patients was prepared for operation by painting with alcoholic solution of iodine (3½ per cent.), and then scrubbing them with a strong sterile solution of hyposulphite of soda until the colour of the iodine had quite disappeared. The patients were under anæsthesia at the time. Scrapings were then taken from the skin thus treated. Four specimens were used in the test for each separate patient. (1) A small amount of the solution of hyposulphite was put into a bouillon tube as a control; (2) skin scrapings taken immediately after the application of the second coat of iodine were put in bouillon tubes; (3) scrapings were taken five minutes after the second coat; (4) scrapings were taken after the decolorisation by hyposulphite solution. At the end of five days' incubation the forty specimens taken from the ten patients showed no traces of bacterial growth.

An attempt was then made to determine whether the sterilising effect penetrated into the deeper layers of the skin. The patient's skin was painted twice with iodine, and under anæsthesia a strip of it four inches long and an inch wide was removed and placed in salt solution. Out of twelve such cases, in five a vigorous culture of *Bacillus subtilis* was rubbed into the skin thirteen to eighteen hours before its removal. A number of bacteriological incubations and experiments were then made, as a result of which it was shown that the salt solution in which the strips of skin were placed was not inhibitory to bacterial growth; that the skin was sterile in practically every case; and that the iodine method killed the *Bacillus subtilis*. The spores of this latter micro-organism are known to be remarkably resistant to antiseptics: they can withstand a 1-1000 solution of perchloride of mercury for an hour.

Dr. Bovee is inclining to the belief that the germicidal power of iodine in sterilising the skin depends more than a little upon the manner of its application. If the coating be made at first thick, before a thin one has been allowed to dry, the penetration will be deeper or the deep penetration more perfect than if the first coating be a thin one. In the latter event the skin recesses may be but partly filled, and when evaporation of the iodine has occurred a thin permeable barrier of iodine is left. Dr. Bovee still stands for the 3½-per-cent. solution, but he is reassured as to the value of the iodine method.

* *The American Journal of Obstetrics*, July 1914, p. 12.

